# Long-term trends of HIV/AIDS incidence in India: an application of joinpoint and age–period–cohort analyses: a gendered perspective

**DOI:** 10.3389/fpubh.2023.1093310

**Published:** 2023-05-16

**Authors:** Neha Shri, Krittika Bhattacharyya, Deepak Dhamnetiya, Mayank Singh, Ravi Prakash Jha, Priyanka Patel

**Affiliations:** ^1^Department of Survey Research and Data Analytics, International Institute for Population Sciences, Mumbai, Maharashtra, India; ^2^Department of Statistics, University of Calcutta, Kolkata, West Bengal, India; ^3^Scientist II (Epidemiology), Clinical Research Unit, All India Institute of Medical Sciences, New Delhi, India; ^4^Department of Fertility and Social Demography, International Institute for Population Sciences, Mumbai, India; ^5^Department of Community Medicine Dr. Baba Saheb Ambedkar Medical College and Hospital, New Delhi, India; ^6^Department of Family & Generations, International Institute for Population Sciences, Mumbai, India

**Keywords:** HIV, incidence, age–period–cohort, GBD, joinpoint regression analysis, India

## Abstract

**Background:**

Monitoring the transmission patterns of human immunodeficiency virus (HIV) in a population is fundamental for identifying the key population and designing prevention interventions. In the present study, we aimed to estimate the gender disparities in HIV incidence and the age, period, and cohort effects on the incidence of HIV in India for identifying the predictors that might have led to changes in the last three decades.

**Data and methods:**

This study utilizes data from the Global Burden of Disease Study for the period 1990–2019. The joinpoint regression analysis was employed to identify the magnitude of the changes in age-standardized incidence rates (ASIRs) of HIV. The average annual percentage changes in the incidence were computed, and the age–period–cohort analysis was performed.

**Results:**

A decreasing trend in the overall estimates of age-standardized HIV incidence rates were observed in the period 1990–2019. The joinpoint regression analysis showed that the age-standardized incidence significantly declined from its peak in 1997 to 2019 (38.0 and 27.6 among males and females per 100,000 in 1997 to 5.4 and 4.6, respectively, in 2019). The APC was estimated to be 2.12 among males and 1.24 among females for the period 1990–2019. In recent years, although the gender gap in HIV incidence has reduced, females were observed to bear a proportionately higher burden of HIV incidence. Age effect showed a decline in HIV incidence by 91.1 and 70.1% among males and females aged between 15–19 years and 75–79 years. During the entire period from 1990–1994 to 2015–2019, the RR of HIV incidence decreased by 36.2 and 33.7% among males and females, respectively.

**Conclusion:**

India is experiencing a decline in new HIV infections in recent years. However, the decline is steeper for males than for females. Findings highlight the necessity of providing older women and young women at risk with effective HIV prevention. This study emphasizes the need for large-scale HIV primary prevention efforts for teenage girls and young women.

## Introduction

Monitoring the spread of HIV in a population is fundamental to controlling HIV in the population in an effort for its prevention and resource allocation. Despite the passing of more than four decades since the onset of the HIV epidemic, HIV continues to pose a challenge to global public health (1). HIV remains a leading cause of mortality in STDs and threatens millions of lives worldwide. Incidence being the key indicator of the rate of HIV transmission in different populations provides the most crucial means of assessing the impact of policies and programs aimed at HIV prevention. Globally, the number of new infections has reduced by 52% since 1997, and in 2020, around 1.5 million people became newly infected with HIV (2). At the national level, ~69,220 new HIV infections occurred in 2019 which has declined by 86% from the peak observed in 1997 (3). The fall in the incidence of new HIV infections has been attributed to changing behaviors (4) and increased awareness of HIV (5). Worldwide, programs to prevent HIV infection have included behavioral targets, such as the adoption of HIV testing, the use of condoms, and a reduction in the number of sex partners. Following the third target of Sustainable Development Goals (SDGs) which aims to end the epidemics of AIDS by 2030 (6), UNAIDS has set a target for each country to reduce the HIV incident cases by 75% between 2010 and 2020 and deaths by 90% between 2010 and 2030 (7). Moreover, gender differences have been observed in the number of new HIV infections, especially at younger ages with the HIV pandemic being “feminized” (8). Low access to information, higher biological susceptibility to HIV, exposure to blood transfusion due to anemia, and pregnancy-related complications coupled with low access to information and treatment make women more vulnerable to HIV (9).

HIV has spread throughout the country after India recorded its first HIV case in the year 1986 (10). In 2019, an estimated 2,349,000 people were living with HIV/AIDS in the country, with an adult prevalence of 0.22%. Furthermore, out of the total number of people living with HIV/AIDS (PLWHA), 3.4% of them were children, whereas ~44% of the total PLWHA aged 15 years and above were female population (3). A glance at India's progress toward the 90-90-90 target of the Joint United Nations Programme on HIV/AIDS (9, 11) suggests that only 79% of HIV-positive people are aware of their HIV status and only 71% of those aware of their HIV status are on HIV treatment (12). Furthermore, the effectiveness of strategies aimed at reducing the number of new infections cannot be determined because of a lack of data on key indicators such as viral suppression rates. Although estimates suggest a decline in the incidence of HIV/AIDS, there exists some interstate variation. For instance, HIV infection rates have risen in recent years in Maharashtra, Bihar, and Uttar Pradesh (3).

To track and monitor the progress made by the country to achieve the specified targets, it is crucial to understand the trend of HIV infection in the previous years. Additionally, indicators, such as incidence rate, are of extreme importance in indicating the effect of interventions and predicting future values for evaluation purposes. Moreover, a trend analysis has advantages in terms of identifying the predictors that may have led to changes within the time frame. Although previous studies have mainly examined the age distribution of HIV incidence or mortality, they have not considered the effects of period and cohort. As a result, it is still unclear what the trends of HIV mortality are in different age groups and what the relative risk (RR) is due to period and cohort effects. Thus, it is imperative to conduct a comprehensive analysis to address these limitations and provide answers to these important questions. This study aims to understand the trend in HIV/AIDS incidence which would help the planners in taking evidence-based actions and would provide a baseline for planning purposes. Additionally, this study addresses gender gaps in the changing incidence rates to have more focused preventive initiatives.

## Materials and methods

### Data source

The GBD study is an important source for comprehending the growing health issues that individuals experience globally in the twenty-first century. The GBD research, led by the Institute for Health Metrics and Evaluation (IHME), is the largest global observational epidemiological study to date. By monitoring progress within and between nations, GBD offers an essential tool to educate medical professionals, researchers, and policymakers, improve lives globally, and raise accountability. The IHME has developed a method for calculating the burden of diseases, injuries, and risk factors to inform health policies and programs throughout the past 20 years. Consistently, GBD provides comparable estimates of the primary disease burden indicators, such as the HIV incidence rate (13).

To determine the mortality rates and cause fractions specific to each cause, the standard Cause of Death Ensemble Model (CODEm) and spatiotemporal Gaussian process regression were used. In recent years, various relevant studies have been reported on the comprehensive description of CODEm (14–17). This method consists of adjustment of cause-specific deaths to match the total all-cause deaths calculated as part of the GBD population, fertility, and mortality estimates and subsequent multiplication of deaths by standard life expectancy at each age to calculate YLLs. Then, for the majority of cases, the Bayesian meta-regression modeling tool DisMod-MR 2.1 was used to guarantee consistency between the incidence, prevalence, remission, excess mortality, and cause-specific mortality. Multiplication of the prevalence estimates was performed by disability weights for the mutually exclusive squeal of diseases and injuries, and thus, YLDs were calculated. The subsequent uncertainty intervals (UIs) were reported for every metric using the 25th and 975th ordered 1,000 draw values of the posterior distribution (13).

The case definition includes HIV having ICD 10 codes B20-B24, C46-C469, and D84.9. Data for the incidence number and rate of HIV in India were extracted from an online tool produced by the IHME, which is publicly available called the Global Health Data Exchange (GHDx) query tool (http://ghdx.healthdata.org/gbd-results-tool). The key sources of data that GBD used include India Demographic and Health Surveys, Medical certification of cause of death of the country and various states, vital statistics, and published scientific articles (13). The present study utilized the Global Burden of Disease Collaborative Network (18) database to systematically summarize and analyze the incidence of HIV and its changes from 1990 to 2019 in India. The annualized rate of change for the rates over the period 1990 to 2019 has been calculated. Age and gender-wise incidence rates have been reported for India.

## Statistical analysis

### Joinpoint regression analysis

The joinpoint regression analysis was employed to identify the changes in ASIRs of HIV among the overall and sex-specific population for all ages by using the joinpoint regression program version 4.5.0.1 (Statistical Research and Applications Branch, National Cancer Institute) (19). This technique (joinpoint regression) differs from traditional piecewise or segmented regression model in terms of identification of joinpoint(s), and their location(s) are estimated inside the model rather than being specified randomly as in the case of piecewise or segmented regression analysis. For every statistically significant segment of the time trend, the model gives the average percentage change (APC) which reflects the rate of change between the two joinpoints. This model also gives the value of average annual percentage change (AAPC) which depicts the overall rate of change in HIV incidence.

The Z test is used to determine whether an AAPC or APC is different from zero. The terms “increase” and “decrease” are used only when the slope (AAPC or APC) of the trend is statistically significant, while the term “stable” refers to a non-significant slope of the trend. For the whole range of our study periods, the average APC (AAPC) is calculated using the best model with a maximum of five joinpoints relating to six segments (20).

### Age–period–cohort analysis

HIV/AIDS incidence rates not only reflect the incidence risk of HIV/AIDS experienced by the population in a given year but also the consolidation of health and wellbeing risks since birth. When estimating the incidence attributed to HIV/AIDS, a common statistical analysis could not decompose these cumulative risks and health hazards (8, 21). The age–period–cohort (APC) analysis is a widely used statistical technique to describe the complex situation of the social, environmental, and historical factors that simultaneously affect individuals and groups of individuals (22). In the current study, APC analysis is used to estimate the net age, period, and cohort effects on the incidence of HIV/AIDS from observed age-specific incidence rates (8, 23–25). It is well-known that APC suffers from an identification problem because of the linear relationship between age, period, and cohort, i.e., cohort = period – age. Therefore, the intrinsic estimator (IE) method was used to decompose the temporal trends and provide unbiased, valid, and relatively efficient estimation results (26, 27). In the APC-IE model, the age-specific incidence rates were recoded into successively 5-year age groups (0–4, 5–9, 10–14, …, 90–94), consecutively 5-year period (1990–94, 1995–99, 2000–04, …, 2015–19), and correspondingly consecutive 5-year birth cohort groups (1900–1904, 1905–1909,..., 2015–2019) to estimate net age, period, and cohort effects on the incidence of HIV/AIDS.

Stata 16.0 software (Stata Corp, College Station, TX, USA) was used to run the APC model. Deviance, Akaike's information criterion (AIC), and the Bayesian information criterion were used to assess the degree of model fitting (BIC). Risk ratios and standard error (SE) coefficients were estimated.

## Results

**Figure 1:**
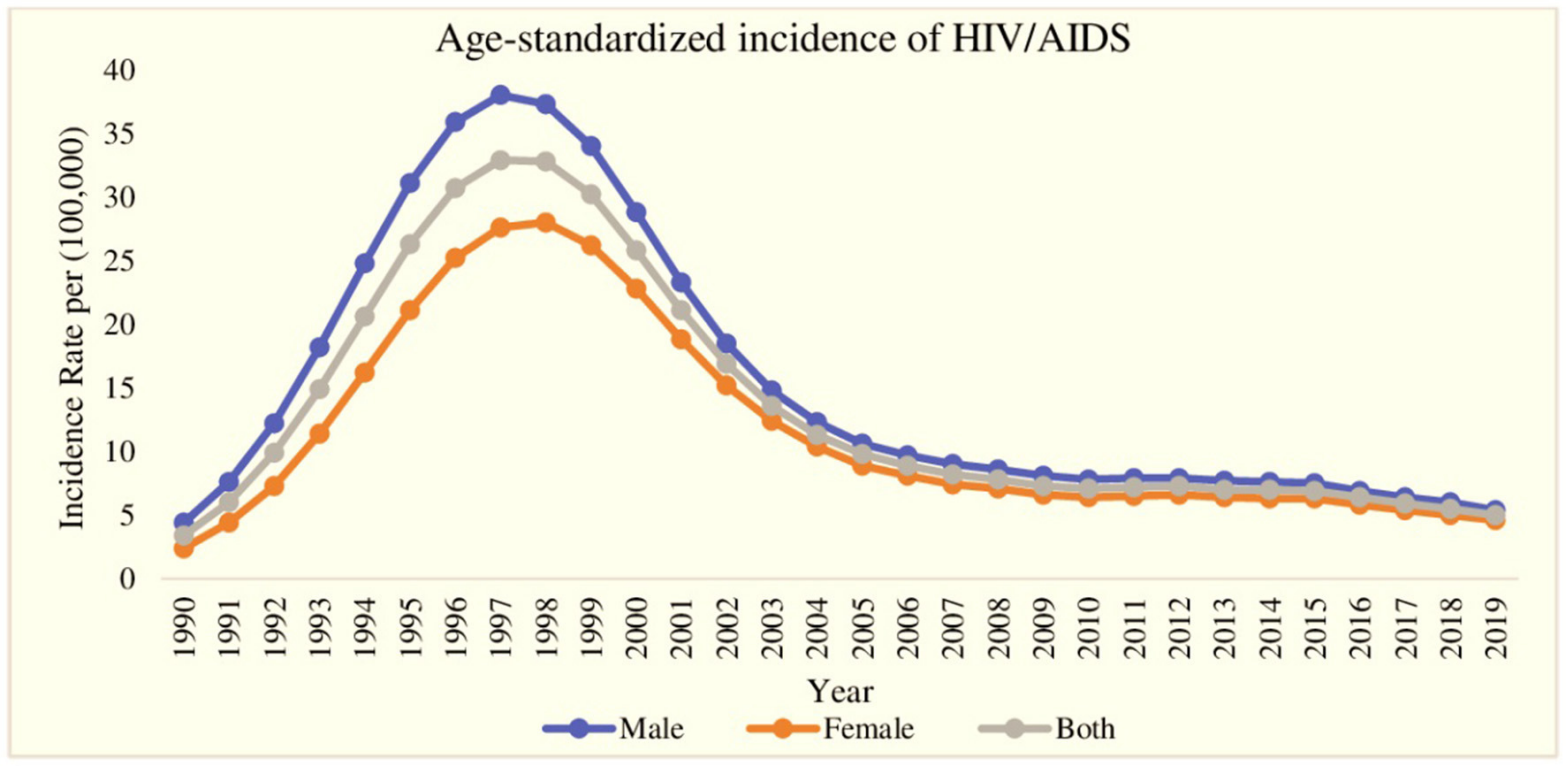
Age-standardized incidence of HIV/AIDS between 1990 and 2019, categorized by gender.

The trend of age-standardized incidence of HIV/AIDS for the period of the last 30 years (1990–2019) is displayed in Figure 1. During the first 7-year period (1990–1997), the age-standardized incidence of HIV/AIDS reported a rapid increase for both genders, and at the same time, the gap in HIV/AIDS incidence among males and females widened with a continuously lower incidence rate in females. After the year 1997, the age-standardized incidence of HIV/AIDS decreased very rapidly till 2009 and reached 5.4 males per 100,000 males and 4.6 females per 100,000 females in 2019. Additionally, in the same period (1997–2019), the gap between male and female HIV/AIDS incidence also decreased. Furthermore, the highest age-standardized incidence of HIV/AIDS was found in the year 1997 with 38.0 cases per 100,000 for males and 27.6 per 100,000 for females.

**Figure 2:**
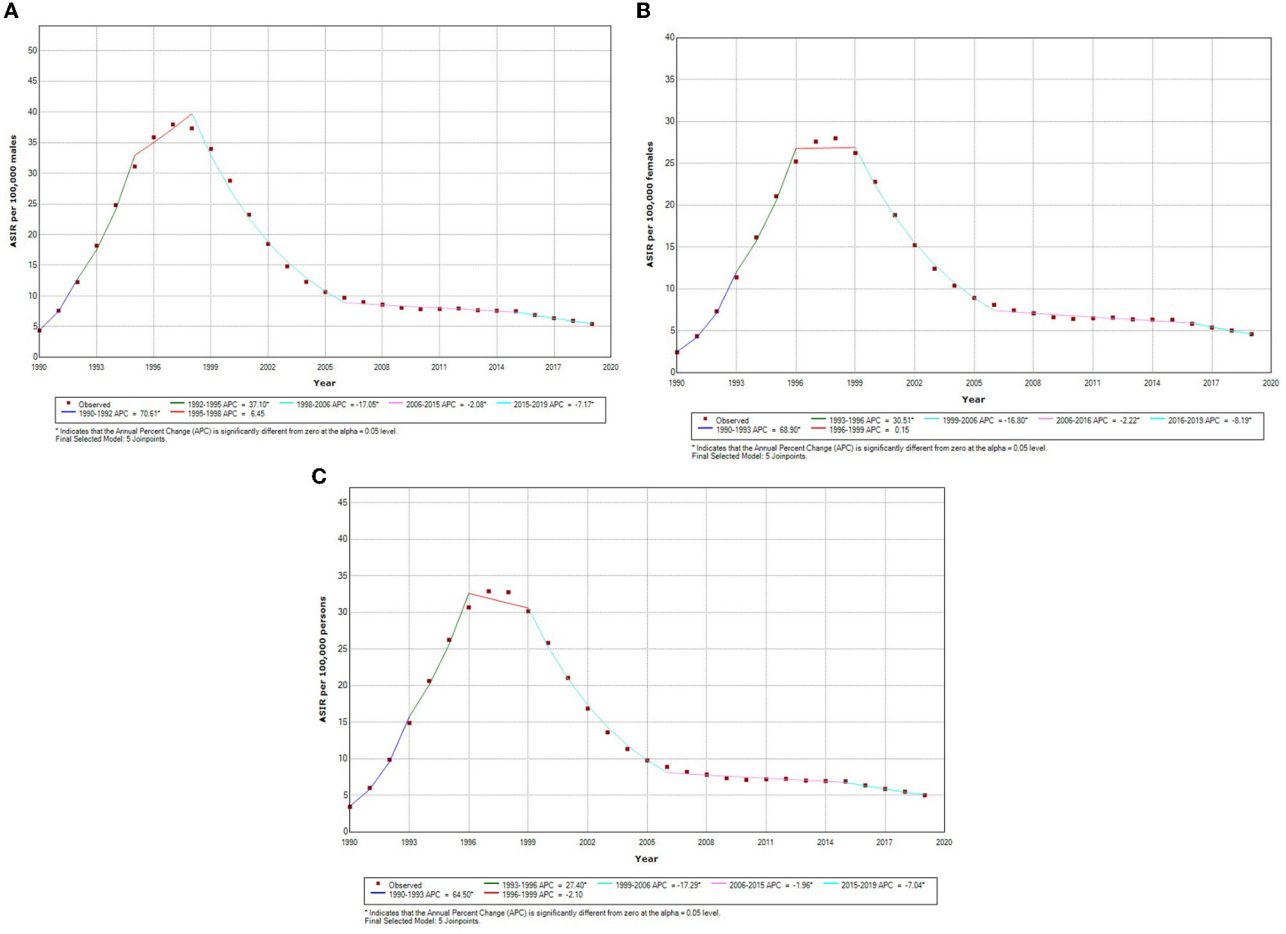
**(A)** Sex-specific temporal trends in age-standardized incidence of incidence male HIV in India based on the jointpoint regression analysis (1990–2019). **(B)** Sex-specific temporal trends in age-standardized incidence of incidence female HIV in India based on the jointpoint regression analysis (1990–2019). **(C)** Sex-specific temporal trends in age-standardized incidence of both sexes HIV in India based on the jointpoint regression analysis (1990–2019). *Indicates that the Annual Percent Change (APC) is significantly different from zero at the alpha = 0.05 level. Final selected model: five joinpoints.

The sex-specific trend of HIV incidence, as shown in Table 1 and Figure 2, was calculated using the joinpoint regression analysis. We know from prior studies that age and gender have a significant influence on HIV incidence; therefore, we have performed gender and age-specific HIV incidence analyses (Table 2, Appendix Figure 1). The column headed with APC shows the incidence trend in each period, whereas the row headed with AAPC shows the overall trend in the entire period of three decades (1990–2019). Within the whole period, ASIR of HIV increased with an AAPC of 0.76 (95% CI: −0.50, 2.05) and 2.12^*^ (95% CI: 0.80, 3.45) among males and females, respectively; however, the overall increase in HIV incidence did not show any significant change, whereas, during the last three decades, increasing and decreasing annual percentage changes have been observed over the six segments as shown in Table 1. For both genders, the highest annual percentage increase in HIV incidence was found in the period 1990–1992 and 1990–1993 with an APC of 70.61 (95% CI: 57.56, 84.75) among males and 68.90 (95% CI: 62.0, 76.09) among females, respectively. Furthermore, among females and males, the highest annual percentage decrease was found in segment 4 of Table 1, with an APC of −17.05^*^ (95% CI: −17.92, −16.16) in males and −16.80 (95% CI: −17.97, −15.62) in females, respectively. The red segment in Figures 2A–C shows the insignificant annual percentage change in the incidence of HIV in all cases, whereas all other segments of Figure 2 show a significant annual percentage change in HIV incidence irrespective of their direction of change.

**Table 1:** Trends in HIV incidence in India from 1990 to 2019 using the joinpoint regression analysis.

**Age standardized incidence rate (male)**			**Age standardized incidence rate (female)**		**Age standardized incidence rate (both sexes)**	
**Segment**	**Year**	**APC** ^*^ **(95% CI)**	**Year**	**APC** ^*^ **(95% CI)**	**Year**	**APC** ^*^ **(95% CI)**
1	1990–1992	70.61^*^ (57.56, 84.75)	1990–1993	68.90^*^ (62.00, 76.09)	1990–1993	64.50^*^ (57.97, 71.31)
2	1992–1995	37.10^*^ (26.61, 48.46)	1993–1996	30.51^*^ (20.07, 41.87)	1993–1996	27.40^*^ (17.48, 38.15)
3	1995–1998	6.45 (−1.70, 15.27)	1996–1999	0.15 (−7.86, 8.87)	1996–1999	−2.10 (−9.73, 6.16)
4	1998–2006	−17.05^*^ (−17.92, −16.16)	1999–2006	−16.80 (−17.97, −15.62)	1999–2006	−17.29^*^ (−18.42, −16.15)
5	2006–2015	−2.08^*^ (−2.92, −1.22)	2006–2016	−2.22^*^ (−2.96, −1.47)	2006–2015	−1.96^*^ (−2.82, −1.09)
6	2015–2019	−7.17^*^ (−9.48, −4.81)	2016–2019	−8.19^*^ (−11.94, −4.28)	2015–2019	−7.04^*^ (−9.39, −4.63)
AAPC^*^	1990–2019	0.76 (−0.50,2.05)	1990–2019	2.12^*^ (0.80, 3.45)	1990–2019	1.24 (−0.02, 2.51)

^*^Indicates that the annual percent change (APC) is significantly different from zero at the alpha = 0.05 level.

APC, annual percentage change; AAPC, average annual percent change; CI, confidence interval.

**Table 2:** Average annual percentage change (AAPC) of HIV incidence in India by age and gender from 1990 to 2019 using the joinpoint regression analysis.

**Age-group (year)**	**AAPC of HIV incidence (95% CI)**	
	**Male**	**Female**
15–19	−0.44 [−1.67, 0.8]	3.09^*^ [1.63, 4.56]
20–24	1.05^*^ [0.15, 1.95]	2.91^*^ [1.86, 3.97]
25–29	0.71 [−0.73, 2.18]	1.89^*^ [0.43, 3.37]
30–34	0.37 [−0.77, 1.52]	1.33^*^ [0.09, 2.59]
35–39	−0.24 [−1.76, 1.3]	0.99 [−0.58, 2.57]
40–44	−1.56 [−3.16, 0.06]	0.02 [−1.33, 1.39]
45–49	−1.88^*^ [−3.25, −0.5]	0.46 [−0.5, 1.43]
50–54	0.69^*^ [0.3, 1.09]	1.51^*^ [0.7, 2.33]
55–59	3.71^*^ [3.18, 4.25]	4.44^*^ [3.9, 4.98]
60–64	3.22^*^ [2.79, 3.66]	3.24^*^ [2.58, 3.9]
65–69	4.30^*^ [3.38, 5.23]	4.92^*^ [4.04, 5.81]
70–74	2.59^*^ [1.58, 3.61]	−0.78 [−2.92, 1.4]
75–79	0.47 [−0.98, 1.93]	−8.31^*^ [−9.77, −6.83]
Overall	0.76 [−0.5, 2.05]	2.12^*^ [0.8, 3.45]

^*^Indicates that the average annual percent change (AAPC) is significantly different from zero at the alpha = 0.05 level.

AAPC, average annual percent change; CI, confidence interval. AIC, BIC, and Deviance were used to assess the degree of model fitness.

Additionally, age-specific joinpoint analysis shows that for some ages, such as 20–24, 50–54, 55–59, 60–64, 65–69, and 70–74 years, men were having significantly positive average annual percentage change irrespective of the fact that overall AAPC value among men does show the significant increase, whereas, at most ages, a significant increase in HIV incidence was observed for females except in the age groups 70–74 and 75–79 years (Table 2). Overall, younger (15–24 years) and older (55–69 years) women experienced a higher burden of HIV incidence in the last three decades (Table 2, Appendix Figure 1).

**Figure 3:**
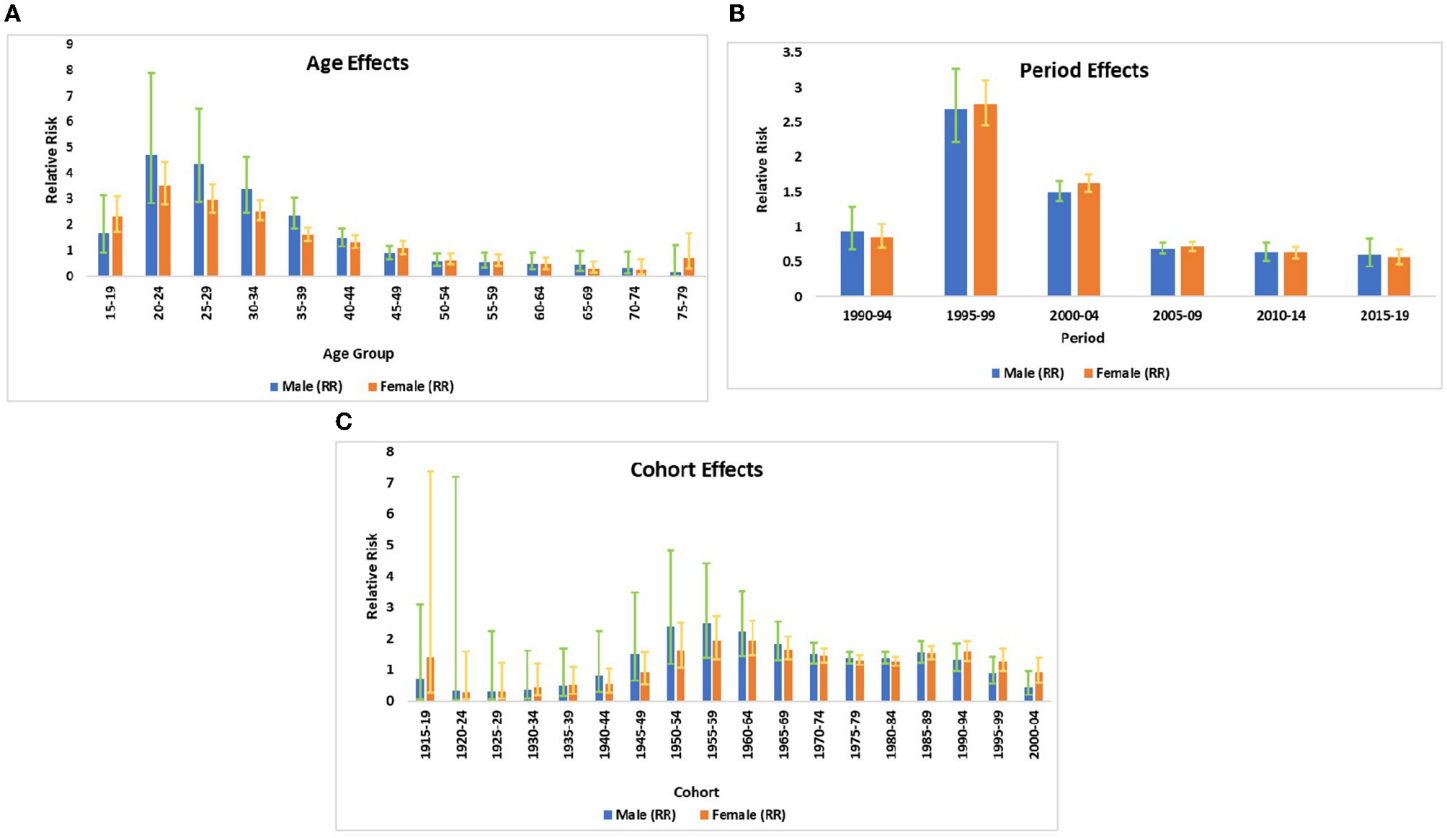
**(A)** The incidence relative risks of HIV/AIDS due to age. **(B)** The incidence relative risks of HIV/AIDS due to period effects. **(C)** The incidence relative risks of HIV/AIDS due to cohort effects.

The relative contribution of age, period, and birth cohort effect on HIV incidence in India is displayed in Figure 3 and Table 3. The APC-IE analysis estimated coefficients for the age, period, and cohort effects (Appendix Table 1). Furthermore, these coefficients were then calculated to their exponential value [exp(coef.) = ecoef.], which denotes the incidence relative risk (RR) at a particular age, period, or birth cohort relative to each average level (Table 3).

**Table 3:** Incidence relative risks of HIV/AIDS due to age, period, and cohort effects.

**Factors**	**Male**		**Female**	
	**RR**	**95% CI**	**RR**	**95% CI**
**Age**				
15–19	1.68	[0.9, 3.15]	2.33	[1.74, 3.12]
20–24	4.72	[2.83, 7.87]	3.51	[2.78, 4.43]
25–29	4.33	[2.89, 6.49]	2.96	[2.45, 3.56]
30–34	3.38	[2.47, 4.62]	2.52	[2.15, 2.94]
35–39	2.37	[1.84, 3.04]	1.59	[1.35, 1.87]
40–44	1.46	[1.15, 1.86]	1.31	[1.08, 1.59]
45–49	0.88	[0.65, 1.19]	1.06	[0.83, 1.36]
50–54	0.59	[0.39, 0.88]	0.62	[0.44, 0.86]
55–59	0.53	[0.32, 0.89]	0.57	[0.38, 0.85]
60–64	0.47	[0.25, 0.9]	0.45	[0.27, 0.73]
65–69	0.42	[0.19, 0.95]	0.28	[0.14, 0.56]
70–74	0.31	[0.1, 0.92]	0.24	[0.09, 0.65]
75–79	0.15	[0.02, 1.21]	0.7	[0.3, 1.68]
**Period**				
1990–94	0.94	[0.69, 1.29]	0.86	[0.71, 1.04]
1995–99	2.69	[2.22, 3.26]	2.76	[2.45, 3.1]
2000–04	1.5	[1.37, 1.65]	1.63	[1.51, 1.76]
2005–09	0.69	[0.62, 0.77]	0.72	[0.66, 0.79]
2010–14	0.63	[0.52, 0.78]	0.63	[0.55, 0.72]
2015–19	0.6	[0.44, 0.83]	0.57	[0.47, 0.69]
**Cohort**				
1915–19	0.72	[0.06, 3.11]	1.41	[0.27, 7.38]
1920–24	0.34	[0.02, 7.19]	0.3	[0.06, 1.6]
1925–29	0.31	[0.04, 2.24]	0.31	[0.07, 1.25]
1930–34	0.36	[0.08, 1.62]	0.45	[0.17, 1.2]
1935–39	0.5	[0.15, 1.68]	0.51	[0.24, 1.09]
1940–44	0.83	[0.3, 2.24]	0.54	[0.28, 1.03]
1945–49	1.51	[0.66, 3.49]	0.92	[0.54, 1.57]
1950–54	2.39	[1.19, 4.81]	1.64	[1.07, 2.5]
1955–59	2.49	[1.4, 4.42]	1.92	[1.35, 2.73]
1960–64	2.24	[1.43, 3.53]	1.94	[1.46, 2.57]
1965–69	1.84	[1.32, 2.56]	1.65	[1.33, 2.06]
1970–74	1.51	[1.21, 1.88]	1.45	[1.23, 1.69]
1975–79	1.37	[1.21, 1.56]	1.31	[1.17, 1.46]
1980–84	1.38	[1.22, 1.56]	1.27	[1.14, 1.41]
1985–89	1.55	[1.25, 1.92]	1.54	[1.33, 1.77]
1990–94	1.33	[0.95, 1.85]	1.57	[1.28, 1.93]
1995–99	0.89	[0.56, 1.42]	1.28	[0.97, 1.7]
2000–04	0.45	[0.21, 0.97]	0.91	[0.59, 1.39]
Deviance	**26,685.52**		**19,234.40**	
AIC	**353.72**		**258.02**	
BIC	**26,493.83**		**19,042.70**	

AIC, BIC, and Deviance were used to assess the degree of model fitness.

### Age effect

Figure 3A displays the relative risk of age effect adjusted by period and birth cohort effects which were found to be statistically significant for both sexes. The result from the age effect shows that the risk of HIV incidence among both genders increased sharply from ages 15–19 to 20–24 years. Thereafter, the risk of HIV incidence started decreasing at a higher pace between the ages of 20–24 and 50–54 years. It was also noted that the risk of HIV incidence among males was relativity higher in comparison with females except for some ages, for instance, females of age 15–19 years were having 1.4 times higher risk of HIV incidence in comparison to male counterparts in the same age strata. Similarly, women of age 75–79 years were at a 4.7 times higher risk of HIV incidence in comparison to males of the same age group. For the net age effect from the 15–19 to 75–79 age group, the risk of HIV incidence decreased by 91.1% among males and 70.1% among females (Table 3).

### Period effect

The relative risk of period effect adjusted by age and birth cohort effect was statistically significant for both sexes in cases of HIV incidence (Figure 3B, Table 2). A sharp increase in the incidence was noted from the period 1990–1994 to 1995–1999. Thereafter, the decreasing risk of HIV incidence was also observed among both sexes from the period 1995–1999 to 2005–2009 followed by a nearly unchanged risk of incidence till 2015–2019. During the entire period from 1990–1994 to 2015–2019, the RR of HIV incidence decreased by 36.2 and 33.7% in males and females, respectively, which indicates the decreasing incidence of HIV among both sexes with an advancing time.

### Cohort effect

The birth cohort effect shows that HIV incidence risk increased in the cohort from 1920–24 to 1955–1959 and subsequently decreasing risk has been noted from 1955–1959 to 1980–1984 in males and females (Figure 3C, Table 3). From the earlier birth cohorts 1915–1919 to more recent birth cohorts 2000–2004, the RR of HIV incidence decreased by 37.5% in males and 35.5% in females.

## Discussion

In monitoring the transmission patterns of disease, designing prevention interventions, and determining the key population for health policy and planning, an accurate estimate of HIV incidence by age and sex is crucial. In the present study, we aimed to estimate the gender disparities in HIV incidence and performed an APC model to estimate the age, period, and cohort effects on the incidence of HIV in India in the period 1990–2019. Our findings demonstrated an increase in HIV incidence in the previous years, i.e., 1990–97, and a considerable decline in new infection rates in the last two decades was observed. These findings are comparable with the trends observed in other regions of the world and India (28, 29). A global study based on GBD reported a decline of 3.0% in age-standardized new HIV infections in the period 2007–2017 (30). India has been working tremendously to eradicate HIV/AIDS and has undertaken various interventions and scaled-up prevention strategies under the NACO in 1992. A comparative study of HIV/AIDS policy reported that different outcomes of HIV/AIDS prevention are mostly due to differences in policy statements, financial stability, and commitments of the government and available healthcare infrastructure (31). Gay men, men who have sex with men, people who use injected drugs, and sex workers are the predominantly affected groups in countries such as the USA, UK, Australia, and India. Integration of family planning and counseling and testing for HIV at health facilities has improved voluntary counseling and testing uptake. For instance, Uganda's national response to HIV/AIDS focusing on risk reduction behavior was responsible for reductions in new HIV infections (32).

Higher incidence rates of the infection were seen among males with the gender gap narrowing down in recent years. Patterns of declining HIV incidence rates were observed across both sexes in the country. With the progress of time, age-standardized incidence rates have been higher among females than males. Furthermore, the results from age-specific joinpoint regression analysis indicated that in the last three decades, women were bearing a significantly higher burden of HIV incidence than men. These findings are in line with the instances from South India and South Africa where a declining incidence of HIV infection was observed; however, the incidence is higher among males than females (28, 33, 34). Yet, it should be noted that a persistent HIV/AIDS epidemic may prompt risk reduction behavior in survivors after the passing of close family members in the family, neighborhood, or community (32). The disproportionate decreases in HIV incidence remain broadly in line with a gender disparity in primary and secondary prevention service utilization in the study region (35). Additionally, it is plausible that men have higher levels of ART coverage which has led to larger declines in the overall incidence (28).

Our findings revealed that the age-specific HIV incidence has decreased over time in all ages; however, the size and timing of this fall differed between ages and sexes. Over time, the age pattern of incidence flattened across ages. The relative risk of incidence peaked at ages 20–24 years for both men and women and then declined subsequently. Surprisingly, women in the age group of 75–79 years were at higher risk of HIV incidence than their male counterparts. Previous studies have highlighted that widowed/divorced/separated women who live alone and those engaged in a high-risk activity are at an increased risk of acquiring HIV and thus require a special provision in older ages. The effect of age-adjusted by period and birth cohort effect indicated that adolescent females (15–19 years) were at higher risk of HIV incidence in comparison to their male counterparts. This suggests that HIV incidence was significantly affected by age among males and females. In line with our findings, a cross-country systematic review and meta-analysis conducted by Birdthistle et al. showed that adolescent females had higher HIV incidence rates than their male peers in several countries (36, 37). Young women are identified to have a higher risk of HIV infection than young men in some settings (30). This has been linked to social and cultural factors such as poverty, less education, and poor access to information deterring young women from accessing sexual and reproductive health services (9). Previous studies conducted across the world have mixed shreds of evidence. Ma et al. reported a higher burden of HIV incidence among women in other parts of the world (38). In contrast, a cohort study in India reported higher incidence rates among men than women (39). This raises the urgent need of promoting health education and raising awareness of HIV prevention among the young population. Changes in the sex structure of the country and higher HIV infections among men are probably the reason for a large gender gap in HIV/AIDS incidence in China and the US (40, 41). Moreover, at higher ages, women aged 45–64 years and the oldest women (75–79 years) were at higher risk of HIV incidence than their male counterparts. Menopause causes vaginal dryness, which encourages micro-injuries that raise the risk of sexually transmitted disease transmission in women aged 50 years and above (42). The occurrence of age-related AIDS alters throughout time as a result of modifying risk factors (e.g., implementation of preventative healthcare programs and changes in individual behavior).

Because period effects can have an impact on various age groups and persons from different birth cohorts throughout different years can also influence the period relationship, interpreting them separately can be challenging (43). The period relative risk of HIV incidence displayed decreasing patterns which are in line with a cross-country study conducted by Martial and colleagues (44). The variations within countries in trends of HIV/AIDS could be explained by the governments' tardy identification of the HIV/AIDS epidemic and the absence or ineffectiveness of control programs that lack political commitment (32). It was observed that the risk of HIV incidence among females was consistently higher than that among males during the period 1995–1999 and 2005–2009 which conforms to the results of other studies (37). The imbalance of power between men and women limits their decision-making capacity to the sexual behavior of their partners often making women more vulnerable to HIV/AIDS (45). Additionally, the adjusted risk of HIV incidence has declined consistently after the period 1995–1999. The authors of a study found a similar decreasing trend in AIDS incidence over the time 1998–1999 (46). Period effects are mostly observed when some changes take place with new interventions being independent of age and cohort (47). Improved healthcare systems, education, correct knowledge regarding ways of HIV transmission, and developed HIV prevention programs might have led to a decline in new HIV infections in the country. The decline in the RRs of HIV incidence can be justified by the fact that India established NACO in the year 1992 and free ART was introduced in the year 2004. The increase in HIV/AIDS awareness campaigns and educational efforts has sparked a conversation about how sexual health might have helped in deflating the HIV incidence in recent years (48). Moreover, flexibility and adoption of new strategies and increase in coverage of services have been part of the government programs.

The cohort effect denoted the declining risk of HIV incidence in the recent birth cohorts for both sexes (44). However, much of this decline took place among males than females. The highest incidence of HIV infection was observed among the 1955–1959 birth cohort among males and the 1960–1964 birth cohort among females. Decreasing rates of incidence of the disease have been observed in people born between 1950 and 1964 in Spain and Brazil (33, 49). The decline in the incidence rates at younger ages is indicative of the effectiveness of prevention options as a national response (32). A cohort-based study conducted in a South African setting also witnessed an early and large decline in HIV incidence among men than women (28). The introduction of a local VMMC program and the scaling up of national testing and counseling services contributed to this decline in Africa. Unprotected sexual intercourse and engagement in sexually risky behavior might have caused an increased risk of HIV among older cohorts (50). In contrast, researchers are of the view that the population in the young age group has much freedom of thought, speech, and choices which makes them more vulnerable to getting hooked to common risk factors of HIV (51). The majority of lifestyle-related risk factors are more evenly distributed among people of the same generation (birth cohort). In recent decades, there have been significant biological and behavioral improvements in HIV prevention, diagnosis, and treatment which can be linked to a decline in the incidence rates in the recent cohorts.

To conclude, our results indicated that India is witnessing a decline in the number of new HIV infections in recent years for males and females. However, the decline is steeper for males than for females. This decline indicates the influence of policy and prevention strategies leading to improvements in the behavioral aspect. Our findings highlight the necessity of providing older women and young women at risk of HIV with effective HIV prevention. Furthermore, a higher incidence rate among young women emphasizes the need for large-scale HIV primary prevention efforts for teenage girls and young women. Since the extensive spread of HIV/AIDS is influenced by socio-economic factors, the reductions in risky behaviors pulled down the incidence rates among younger ages.

## Data availability statement

The datasets presented in this study can be found in online repositories. The names of the repository/repositories and accession number(s) can be found at: https://vizhub.healthdata.org/gbd-results/.

## Author contributions

NS, MS, and PP contributed in conceptualizing the study. NS, KB, DD, MS, RJ, and PP were responsible for the analysis. All authors contributed to the interpretation of the data, critically revised all versions of the manuscript, and approved the final version.
