# Supplementary material for: Long-term trends of HIV/AIDS incidence in India: an application of joinpoint and age–period–cohort analyses: a gendered perspective
Source: Front Public Health. 2023 May 16;11:1093310. doi: 10.3389/fpubh.2023.1093310 (PMC10227429; doi:10.3389/fpubh.2023.1093310)
Supplement: Supplementary file 1 [file Table_1.docx]

**Appendix Table 1:** HIV/AIDS incidence rates estimated coefficients for the age, period and cohort effects

| Factors | Male | | | Female | | |
| --- | --- | --- | --- | --- | --- | --- |
|  | **Coef** | **95% CI** |  | **Coef** | **95% CI** |  |
| Age | | | | | | |
| 15-19 | 0.521503 | -0.10598 | 1.148983 | 0.844943 | 0.551481 | 1.138405 |
| 20-24 | 1.552026 | 1.041295 | 2.062757 | 1.256207 | 1.023566 | 1.488848 |
| 25-29 | 1.464811 | 1.059557 | 1.870066 | 1.083935 | 0.897978 | 1.269892 |
| 30-34 | 1.216679 | 0.903632 | 1.529725 | 0.922667 | 0.766006 | 1.079327 |
| 35-39 | 0.861668 | 0.611936 | 1.1114 | 0.465264 | 0.302656 | 0.627872 |
| 40-44 | 0.379251 | 0.137244 | 0.621258 | 0.272566 | 0.080344 | 0.464789 |
| 45-49 | -0.12937 | -0.42869 | 0.169961 | 0.061762 | -0.18157 | 0.305095 |
| 50-54 | -0.5324 | -0.93483 | -0.12997 | -0.48548 | -0.81932 | -0.15164 |
| 55-59 | -0.62929 | -1.14415 | -0.11442 | -0.56403 | -0.96499 | -0.16307 |
| 60-64 | -0.75239 | -1.39744 | -0.10735 | -0.80765 | -1.29656 | -0.31875 |
| 65-69 | -0.85874 | -1.66092 | -0.05656 | -1.27613 | -1.97069 | -0.58156 |
| 70-74 | -1.1685 | -2.25876 | -0.07823 | -1.42391 | -2.41436 | -0.43345 |
| 75-79 | -1.92525 | -4.04154 | 0.191034 | -0.35016 | -1.22074 | 0.520427 |
| Period | | | | | | |
| 1990-94 | -0.05852 | -0.37164 | 0.2546 | -0.1504 | -0.33872 | 0.03793 |
| 1995-99 | 0.990204 | 0.7985 | 1.181908 | 1.01413 | 0.896925 | 1.131336 |
| 2000-04 | 0.406532 | 0.313455 | 0.499609 | 0.487902 | 0.410948 | 0.564855 |
| 2005-09 | -0.3708 | -0.47906 | -0.26253 | -0.32312 | -0.41651 | -0.22973 |
| 2010-14 | -0.45852 | -0.66219 | -0.25484 | -0.46103 | -0.5946 | -0.32746 |
| 2015-19 | -0.5089 | -0.82778 | -0.19002 | -0.56749 | -0.76117 | -0.37381 |
| Cohort | | | | | | |
| 1915-19 | -0.33076 | -2.79514 | 1.133616 | 0.340369 | -1.31843 | 1.999162 |
| 1920-24 | -1.07941 | -4.13197 | 1.973146 | -1.20128 | -2.87453 | 0.471963 |
| 1925-29 | -1.17267 | -3.15169 | 0.806344 | -1.18174 | -2.59041 | 0.226929 |
| 1930-34 | -1.01488 | -2.50924 | 0.479475 | -0.7984 | -1.78213 | 0.185328 |
| 1935-39 | -0.69439 | -1.90931 | 0.520526 | -0.66668 | -1.41883 | 0.085475 |
| 1940-44 | -0.19162 | -1.18816 | 0.804919 | -0.62118 | -1.26759 | 0.025228 |
| 1945-49 | 0.414867 | -0.41906 | 1.248799 | -0.08552 | -0.62017 | 0.449132 |
| 1950-54 | 0.873154 | 0.175905 | 1.570402 | 0.494209 | 0.070947 | 0.917472 |
| 1955-59 | 0.912393 | 0.338953 | 1.485834 | 0.652699 | 0.302561 | 1.002838 |
| 1960-64 | 0.80786 | 0.355672 | 1.260048 | 0.660176 | 0.377932 | 0.94242 |
| 1965-69 | 0.608307 | 0.274715 | 0.9419 | 0.50374 | 0.286221 | 0.721259 |
| 1970-74 | 0.409293 | 0.18948 | 0.629107 | 0.36823 | 0.210935 | 0.525525 |
| 1975-79 | 0.317636 | 0.190171 | 0.445102 | 0.268359 | 0.156003 | 0.380716 |
| 1980-84 | 0.323234 | 0.199008 | 0.447459 | 0.238953 | 0.13244 | 0.345466 |
| 1985-89 | 0.439672 | 0.225745 | 0.653598 | 0.429025 | 0.284431 | 0.57362 |
| 1990-94 | 0.282269 | -0.0486 | 0.613138 | 0.450348 | 0.245026 | 0.65567 |
| 1995-99 | -0.11351 | -0.57804 | 0.35102 | 0.247831 | -0.03231 | 0.52797 |
| 2000-04 | -0.79144 | -1.54759 | -0.03529 | -0.09913 | -0.53105 | 0.332785 |
