# Supplementary material for: Long-term trends of HIV/AIDS incidence in India: an application of joinpoint and age–period–cohort analyses: a gendered perspective
Source: Front Public Health. 2023 May 16;11:1093310. doi: 10.3389/fpubh.2023.1093310 (PMC10227429; doi:10.3389/fpubh.2023.1093310)
Supplement: Supplementary file 2 [file Data_Sheet_1.docx]

**Long-term trends of HIV/AIDS incidence in India: An application of joinpoint and Age Period Cohort Analysis: Gendered perspective**

**Appendix Figure 1:** Sex-specific temporal trends in age standardised incidence of HIV/AIDS in India based on the joinpoint regression analysis (1990–2019). Note: Figure A to M shows the male HIV incidence in increasing order of age group from 15-19 to 75-79 years. Figure I to XIII shows the female HIV incidence in increasing order of age group from 15-19 to 75-79 years. Figure N and XIV shows the overall HIV incidence among males and females respectively based on joinpoint regression analysis.

| A  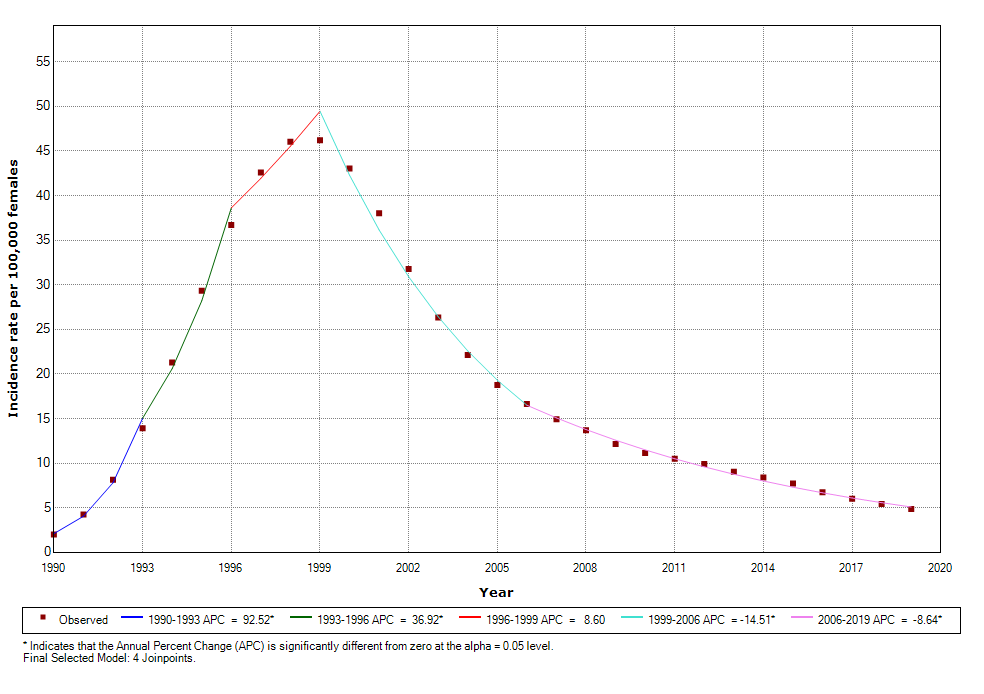 | B  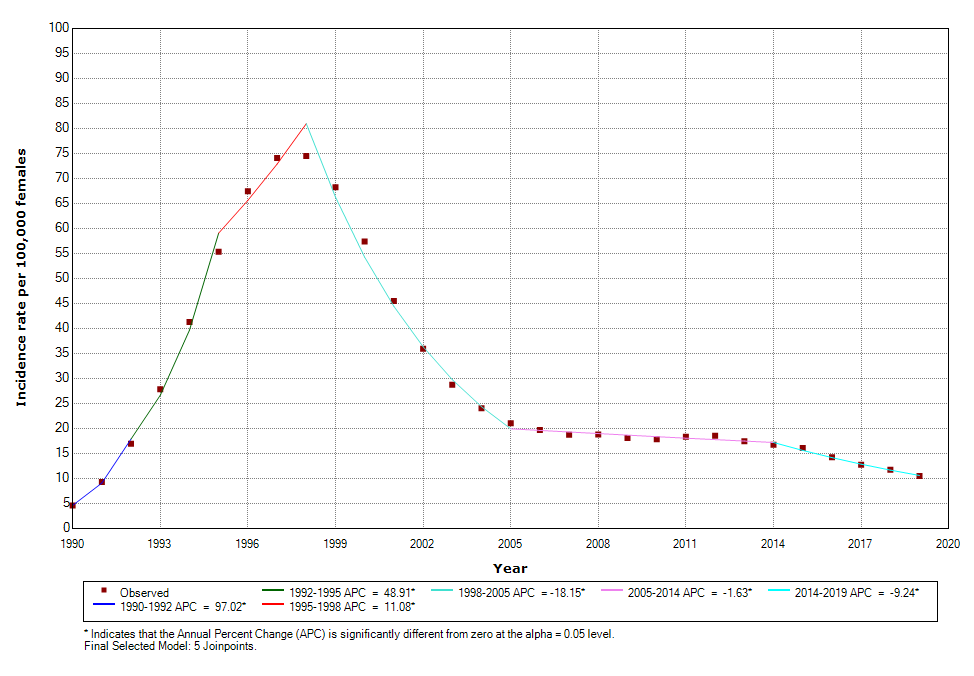 | C  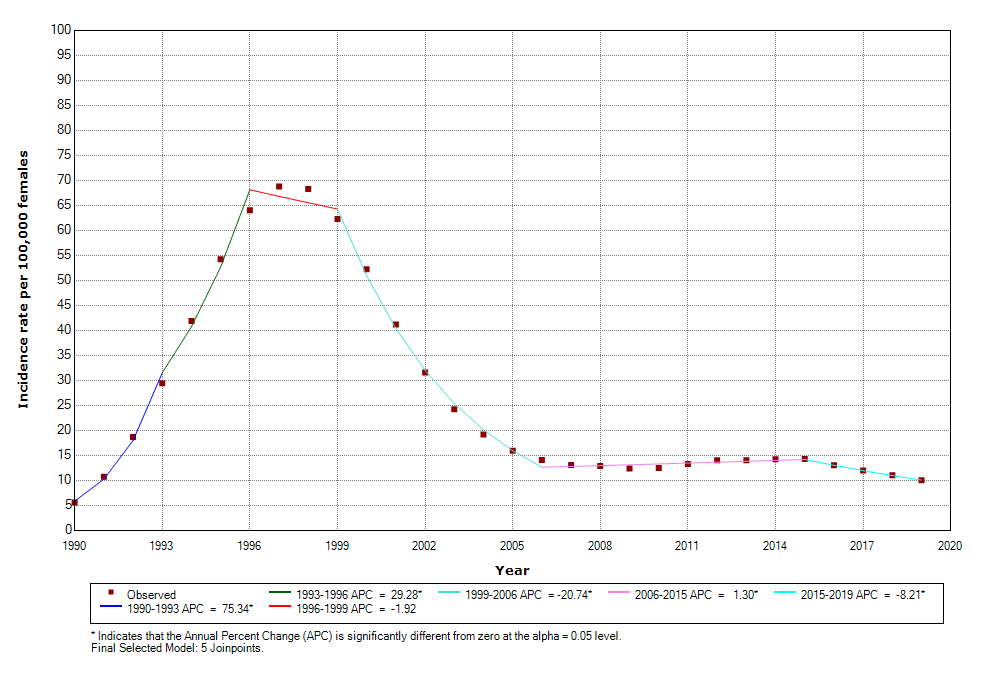 |
| --- | --- | --- |
| D  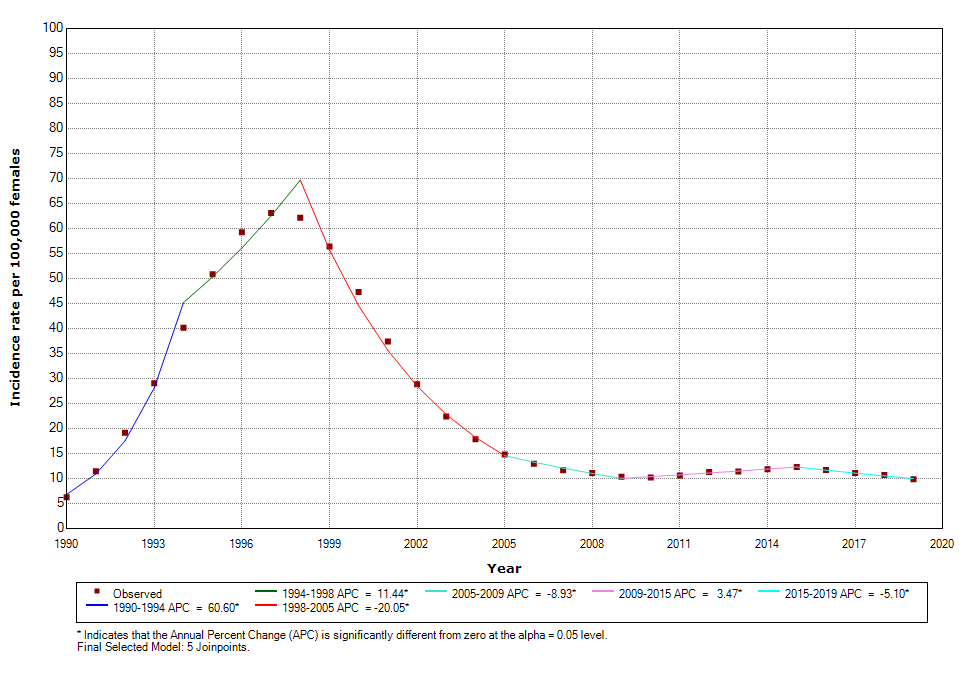 | E  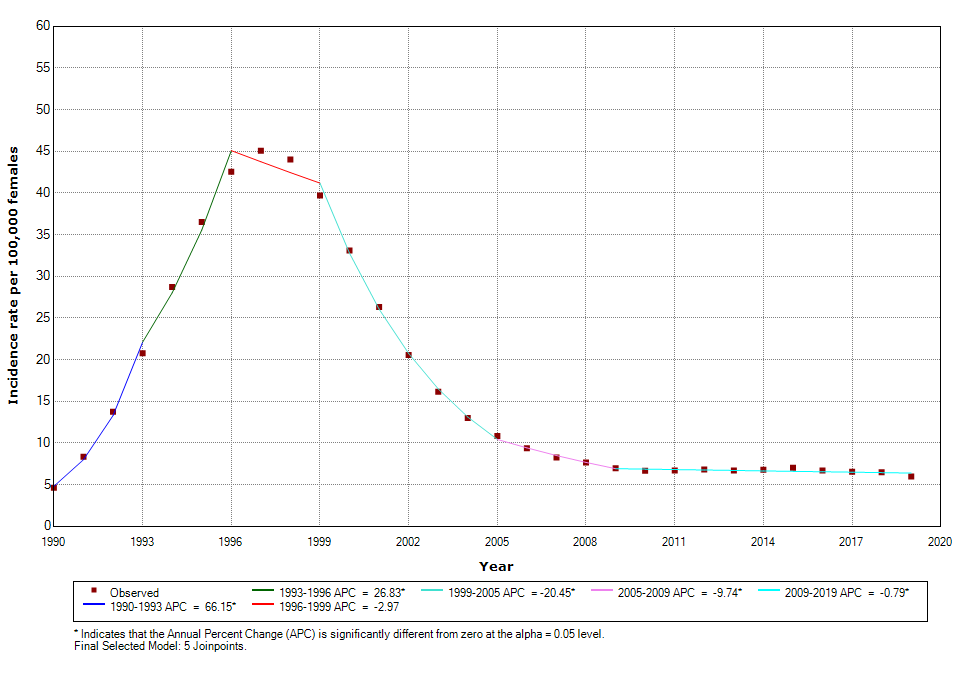 | F  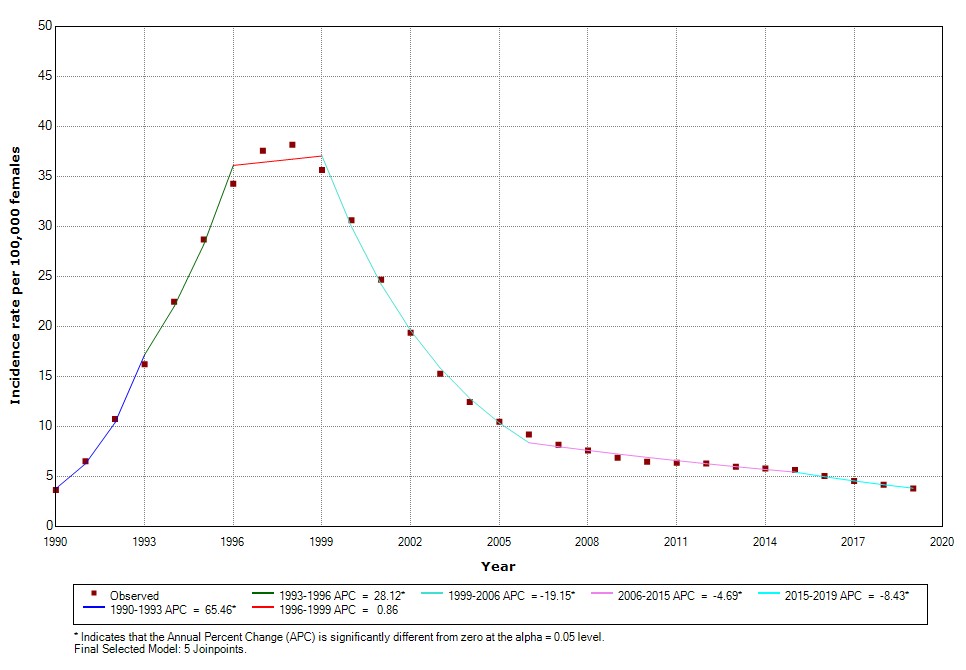 |
| G  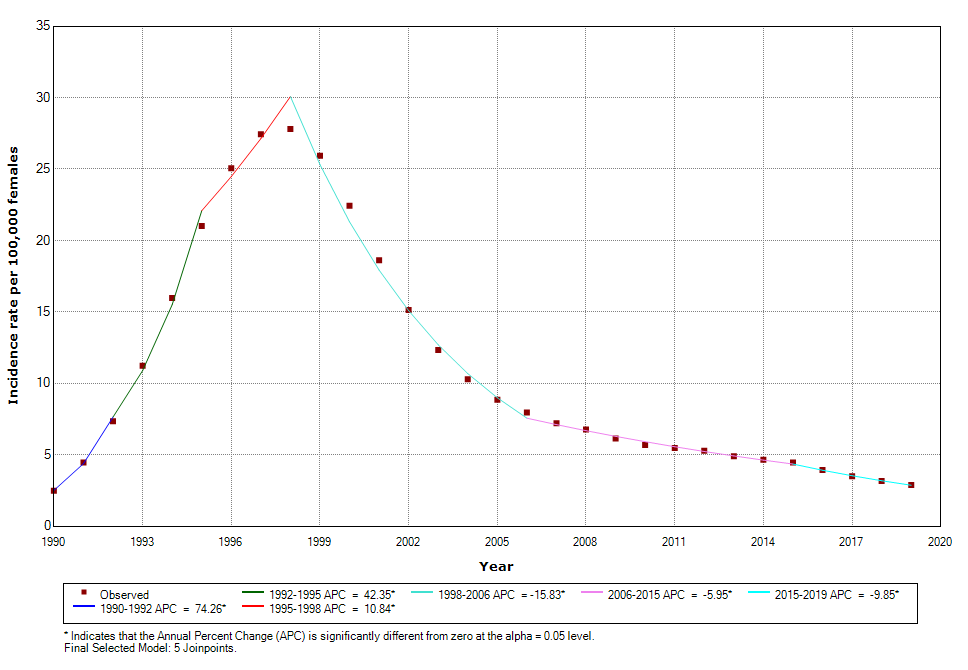 | H  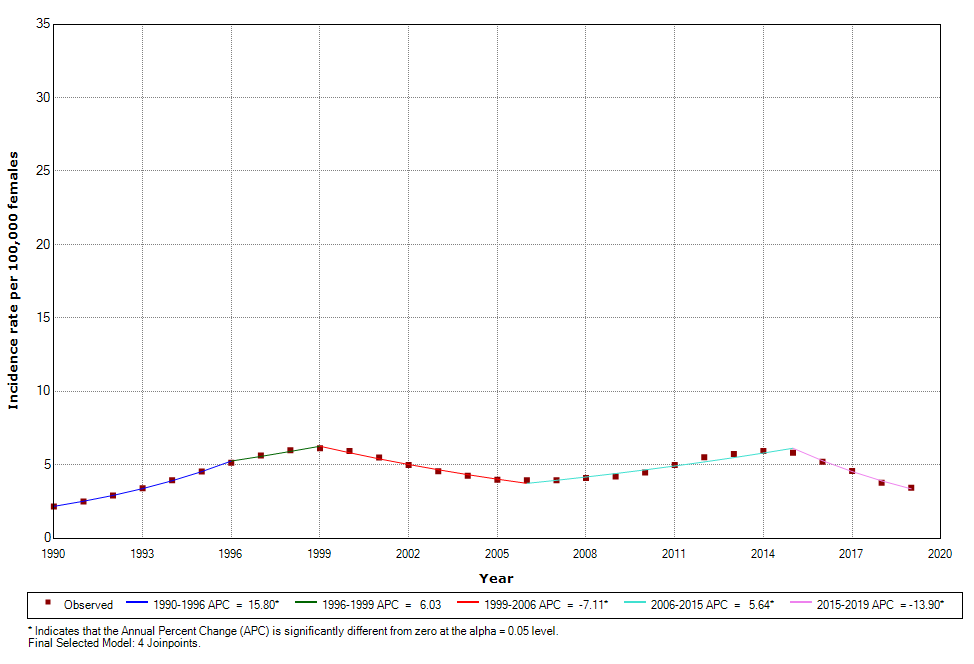 | I  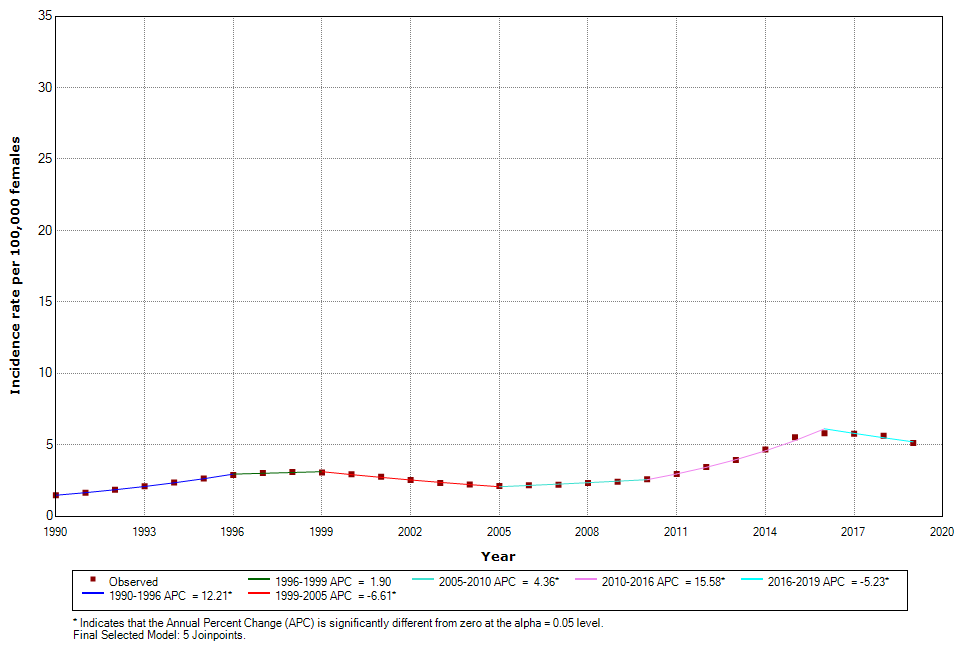 |
| J  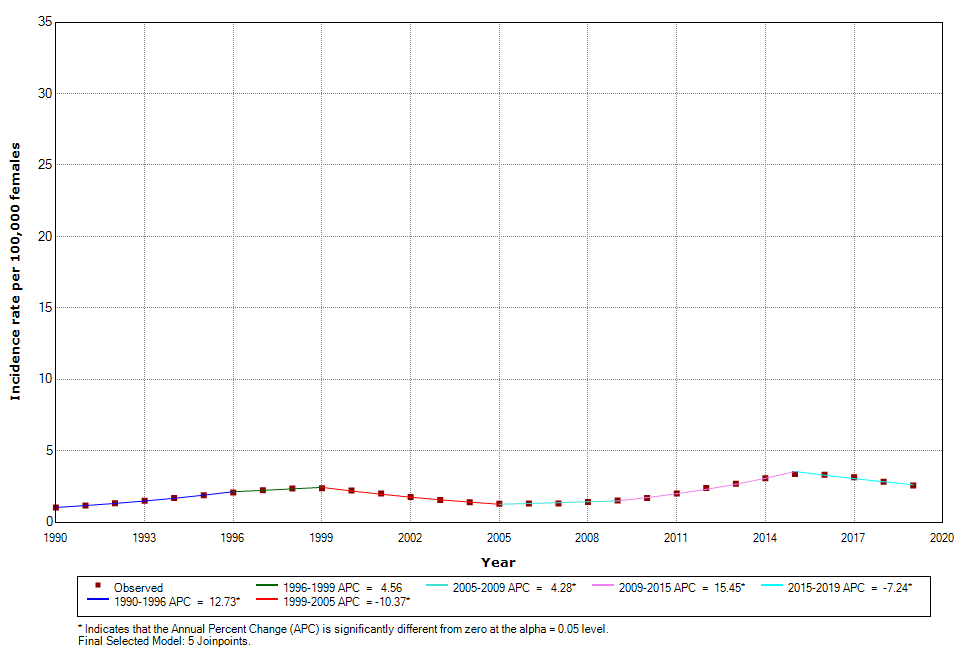 | K  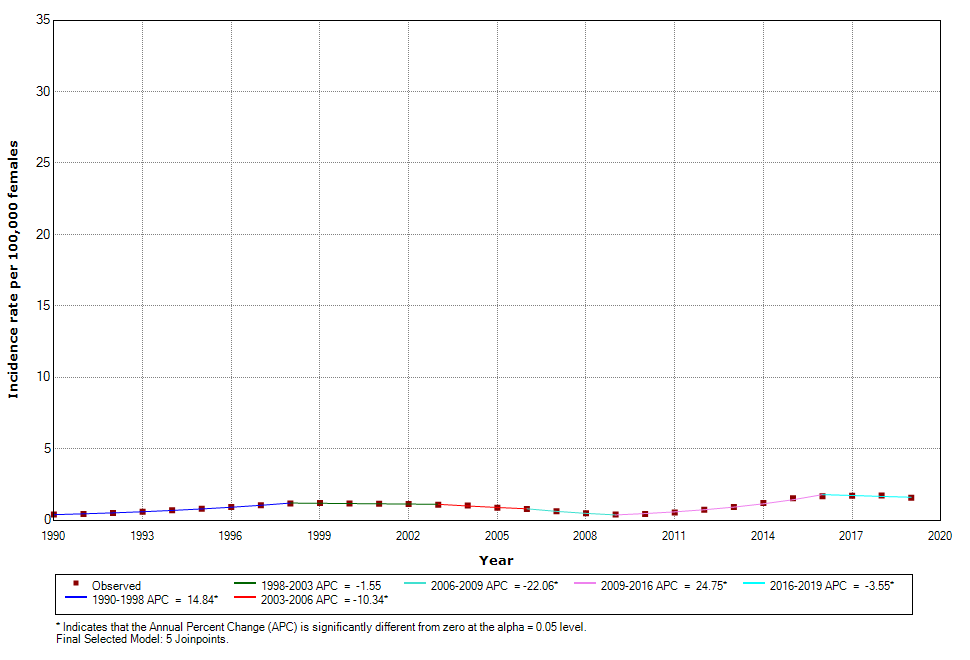 | L  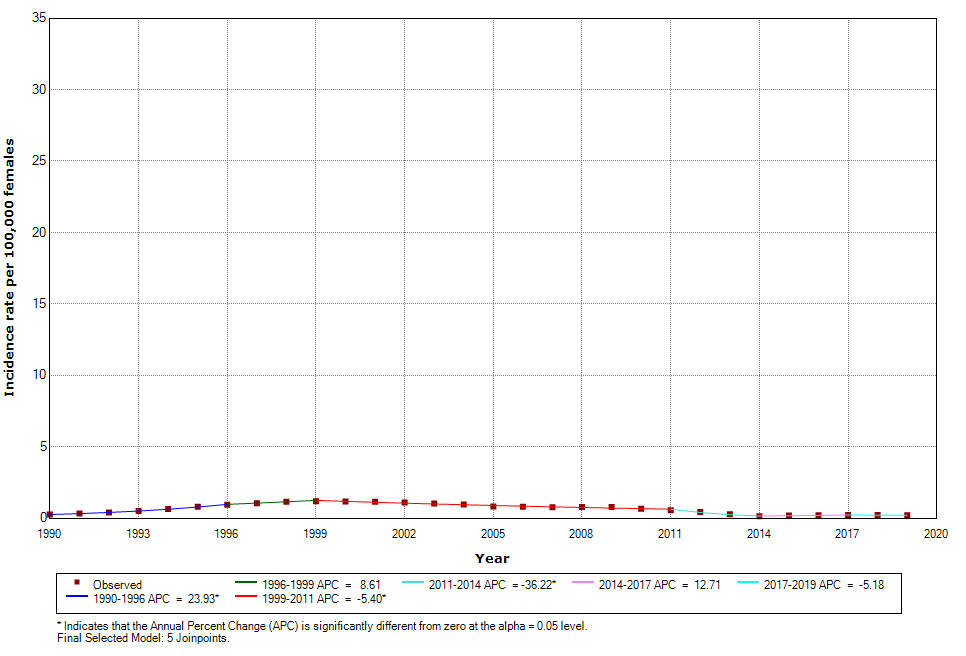 |
| M  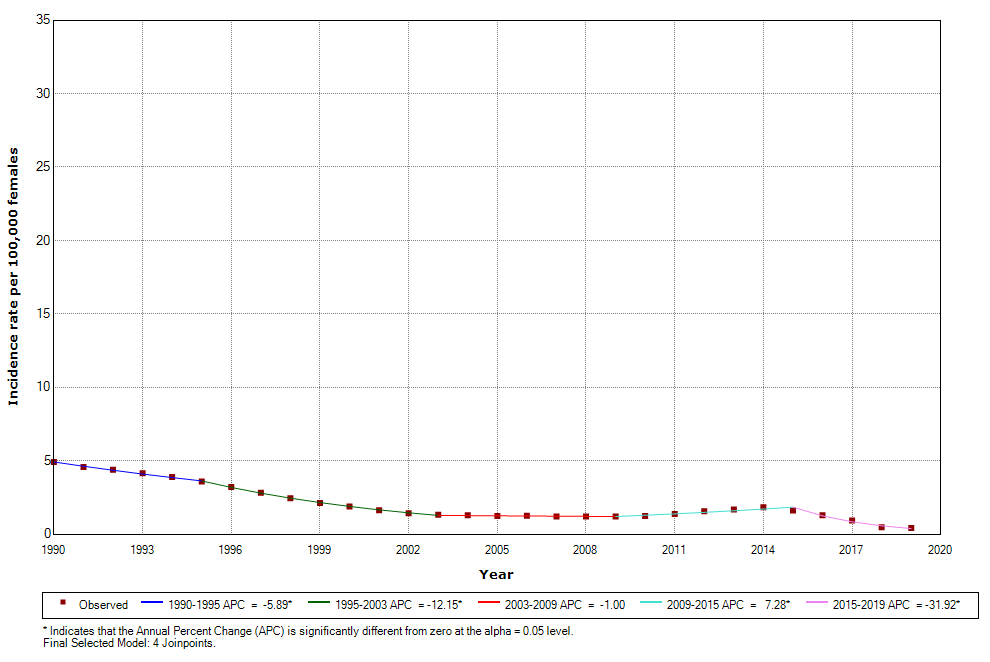 | N  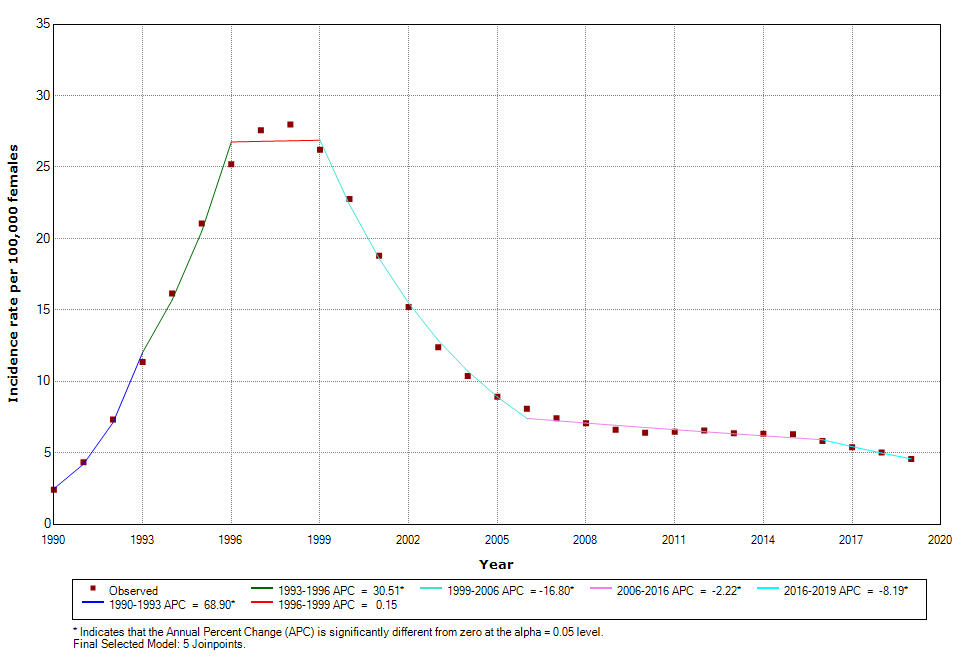 |  |
| I  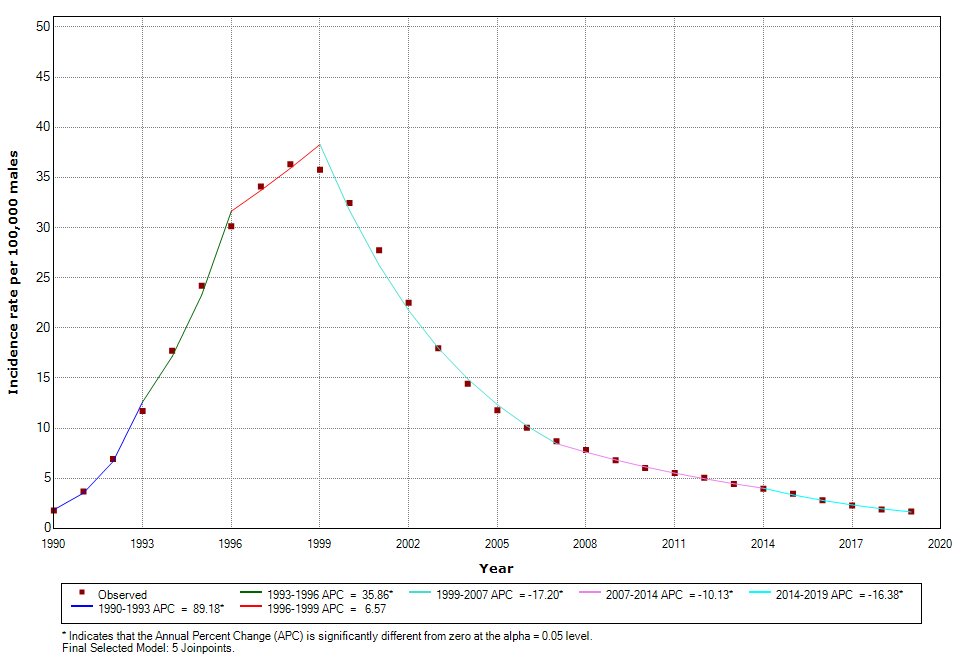 | II  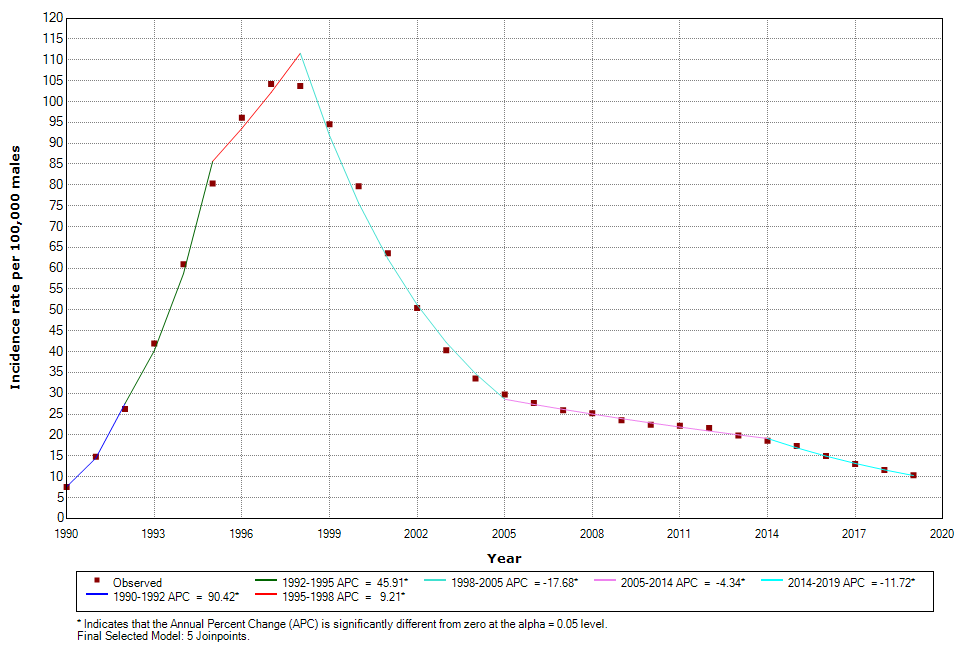 | III  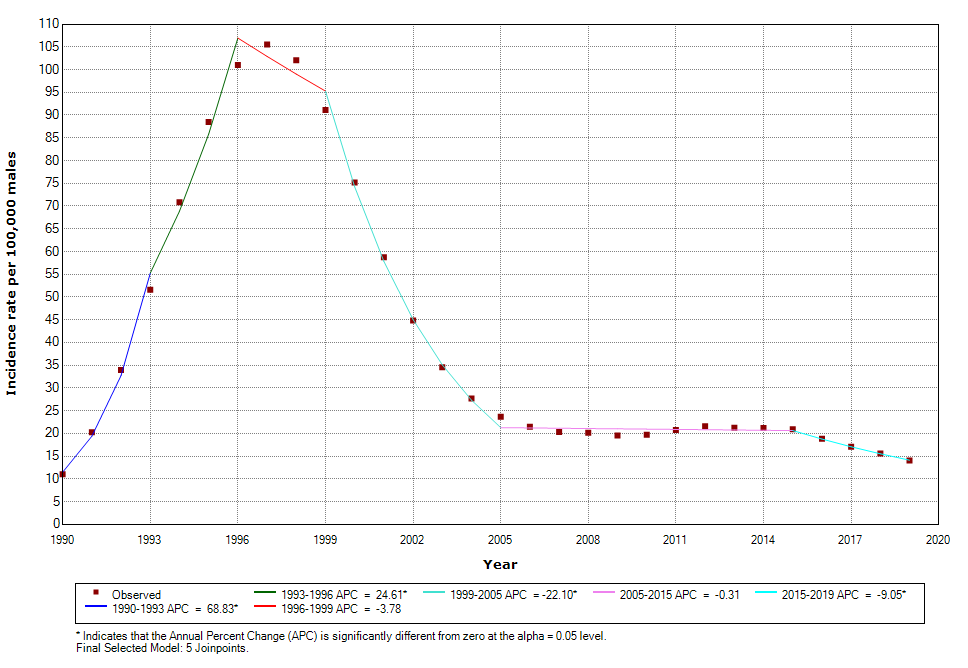 |
| IV  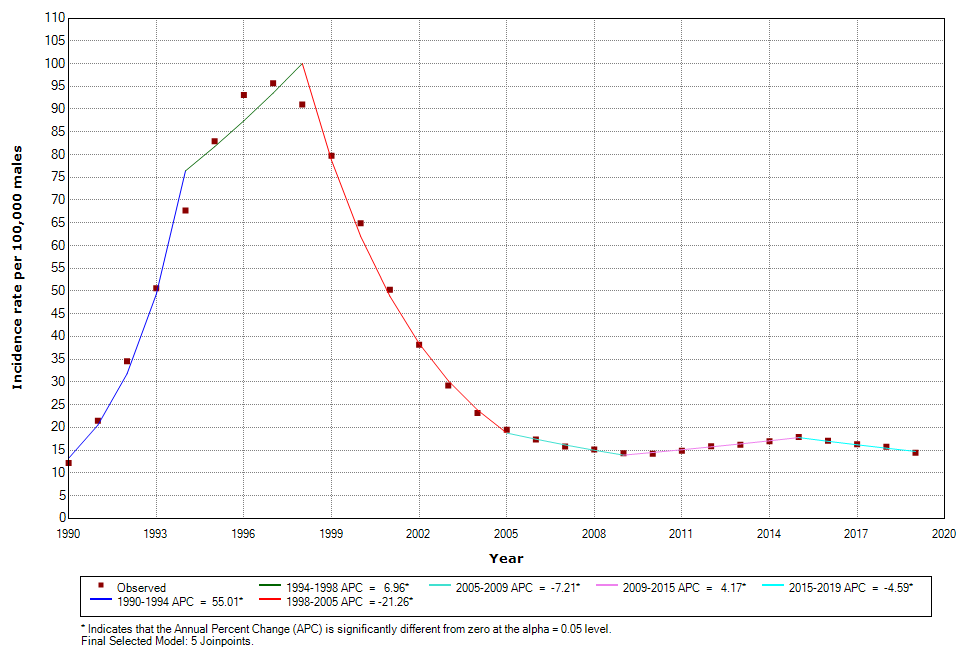 | V  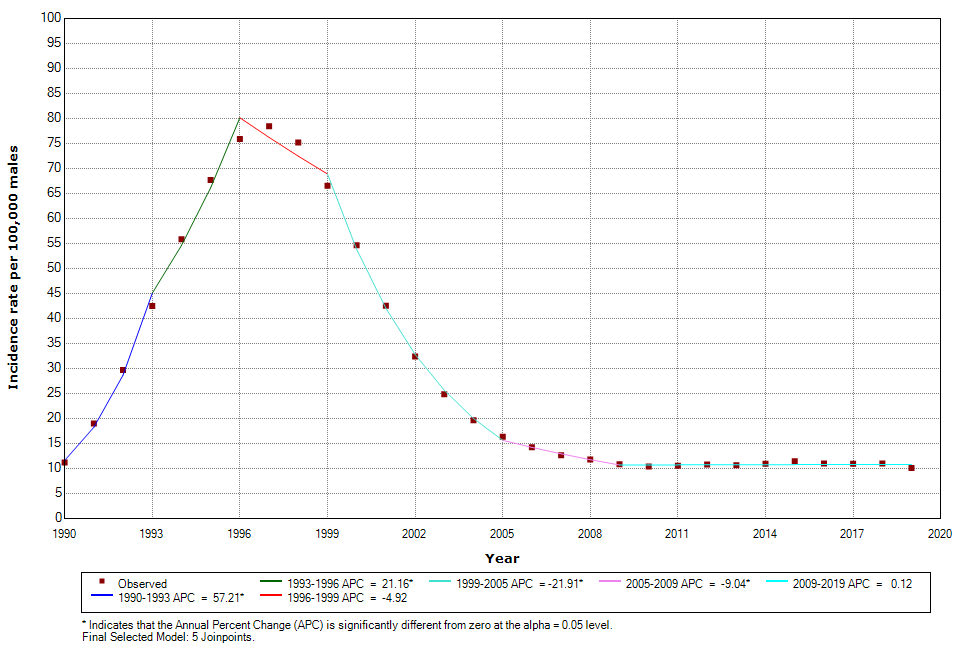 | VI  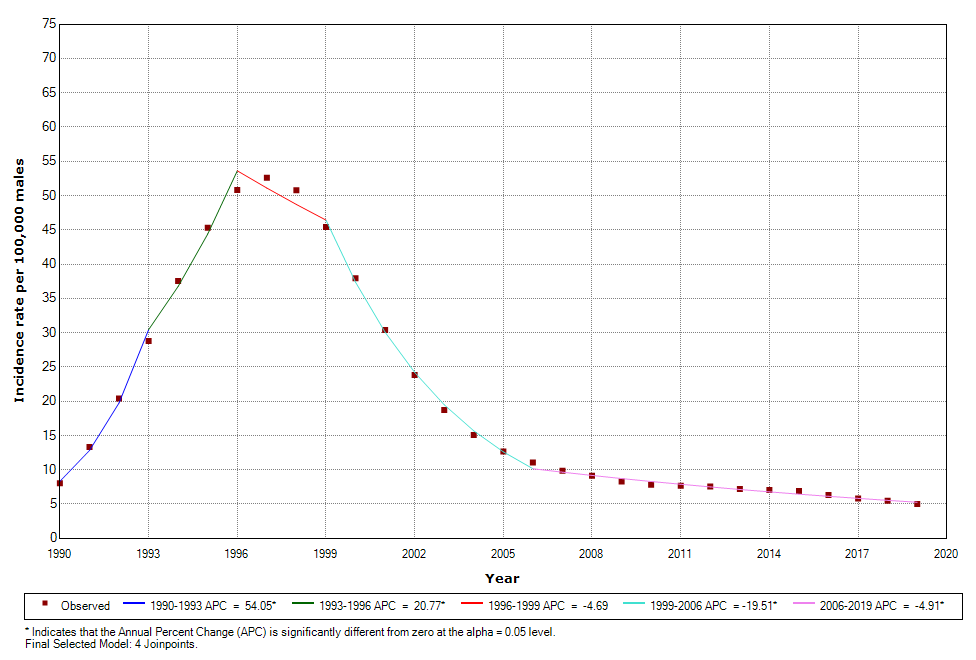 |
| VII  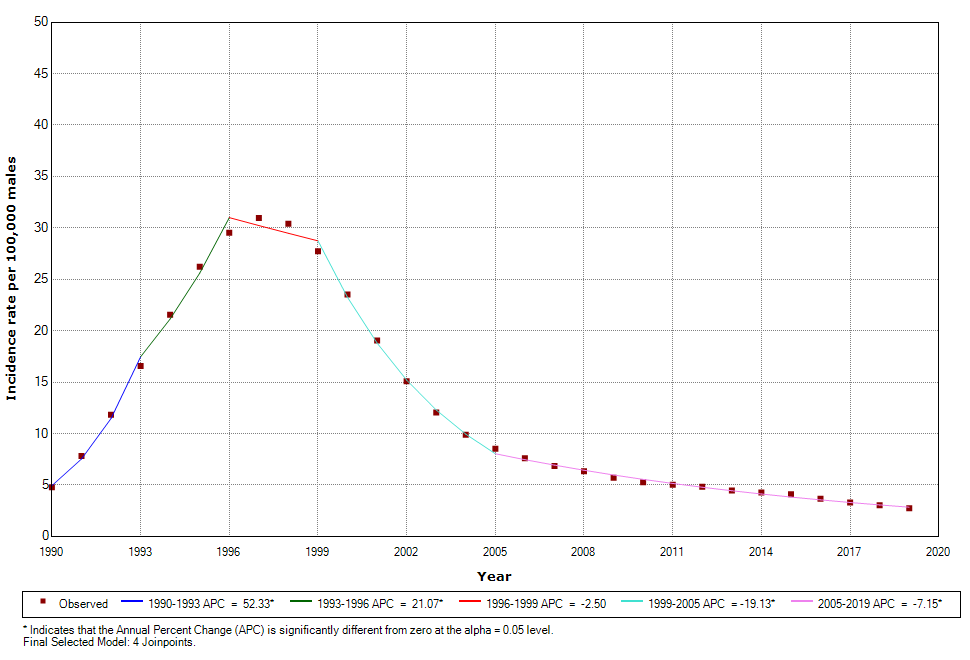 | VIII  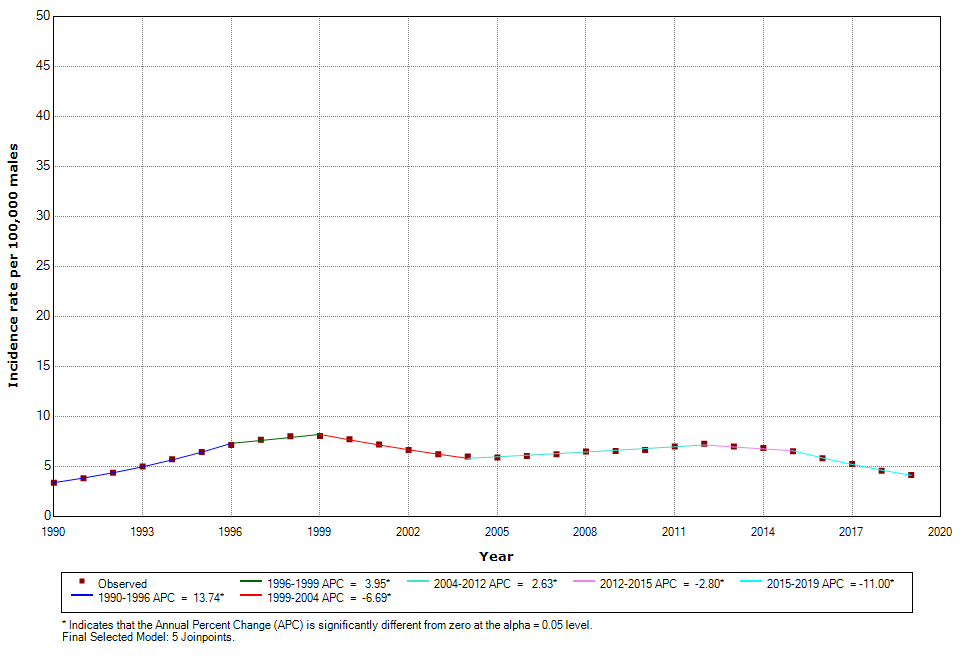 | IX  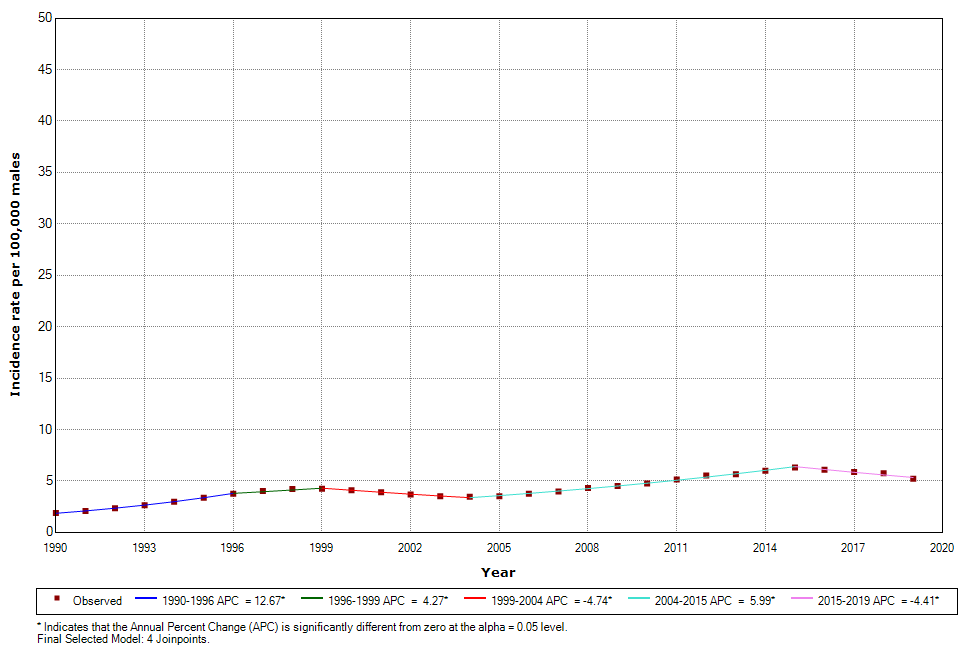 |
| X  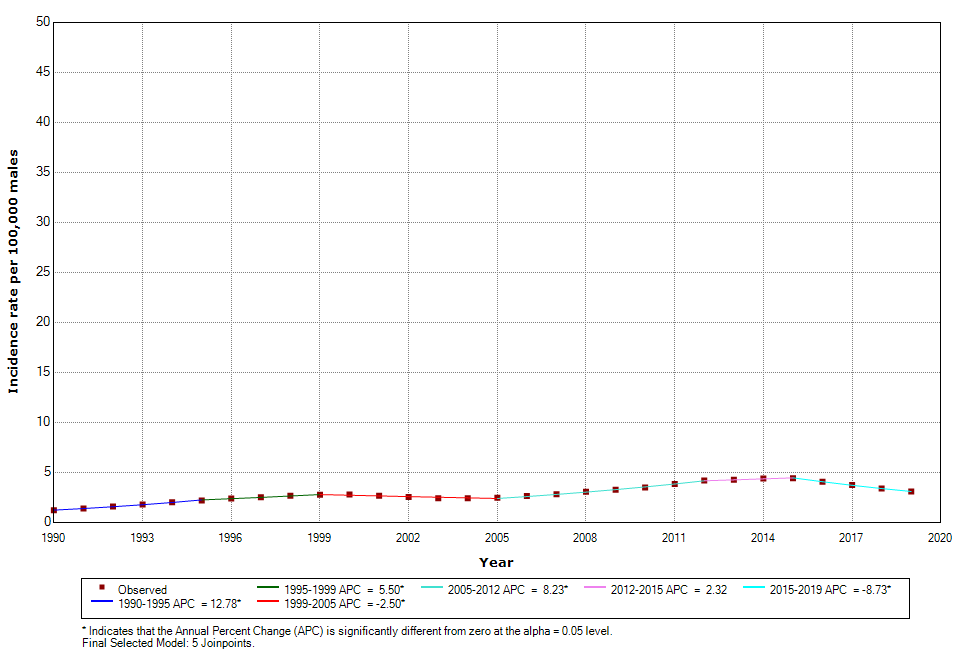 | XI  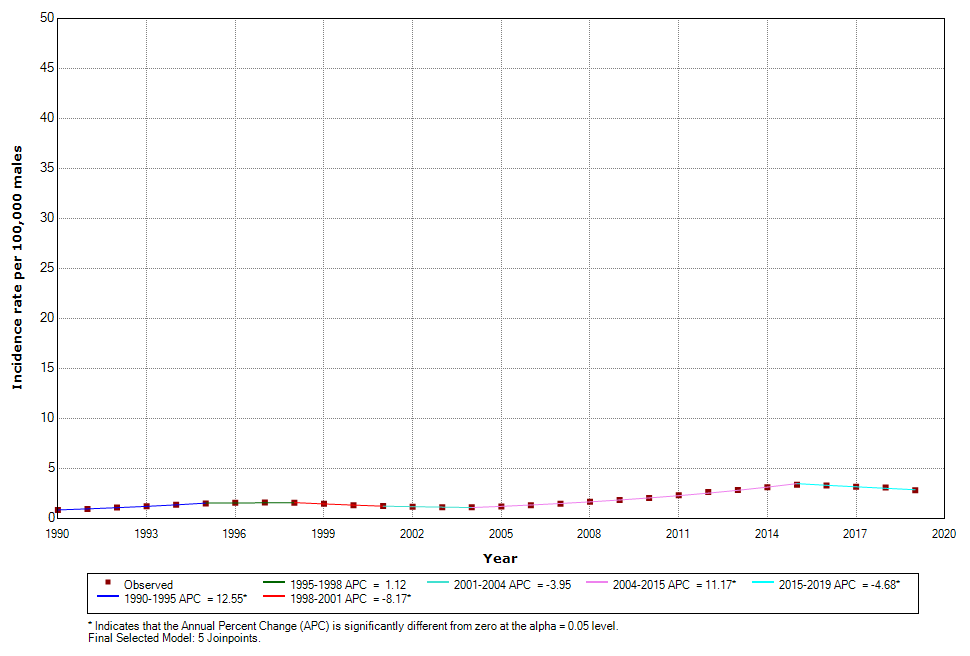 | XII  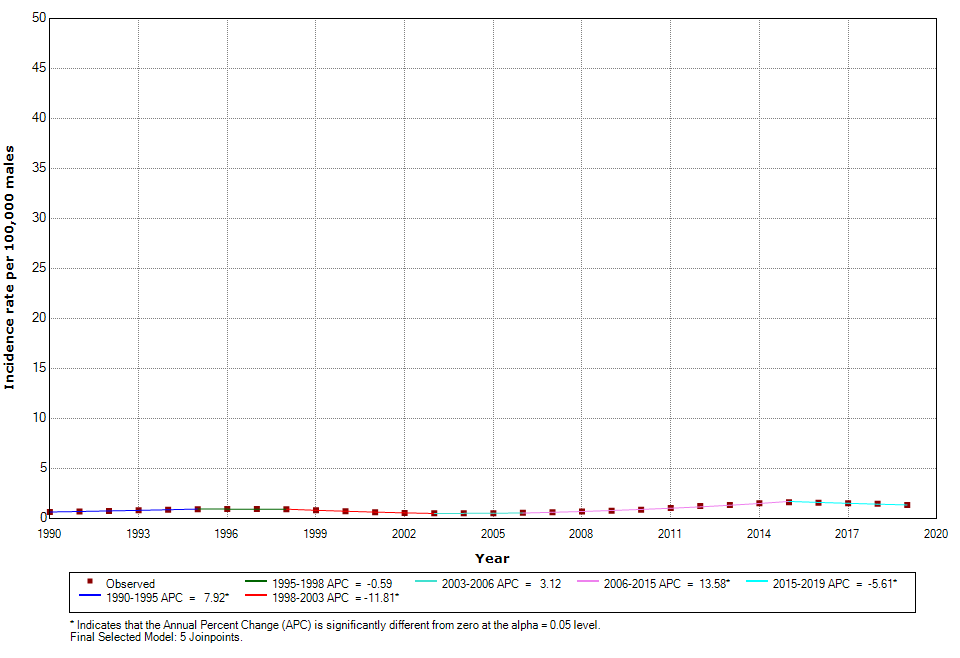 |
| XIII  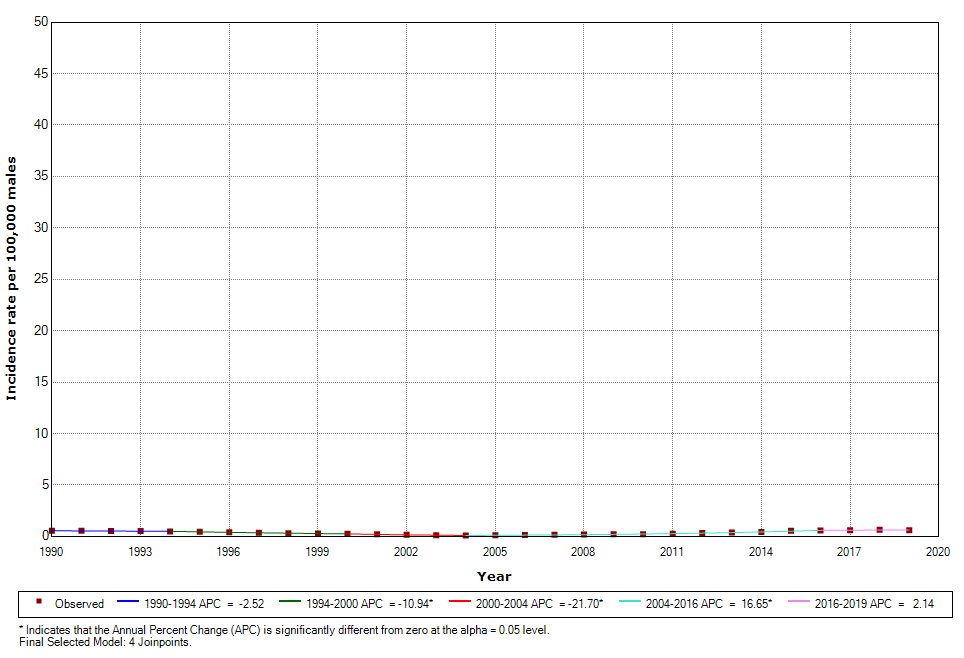 | XIV  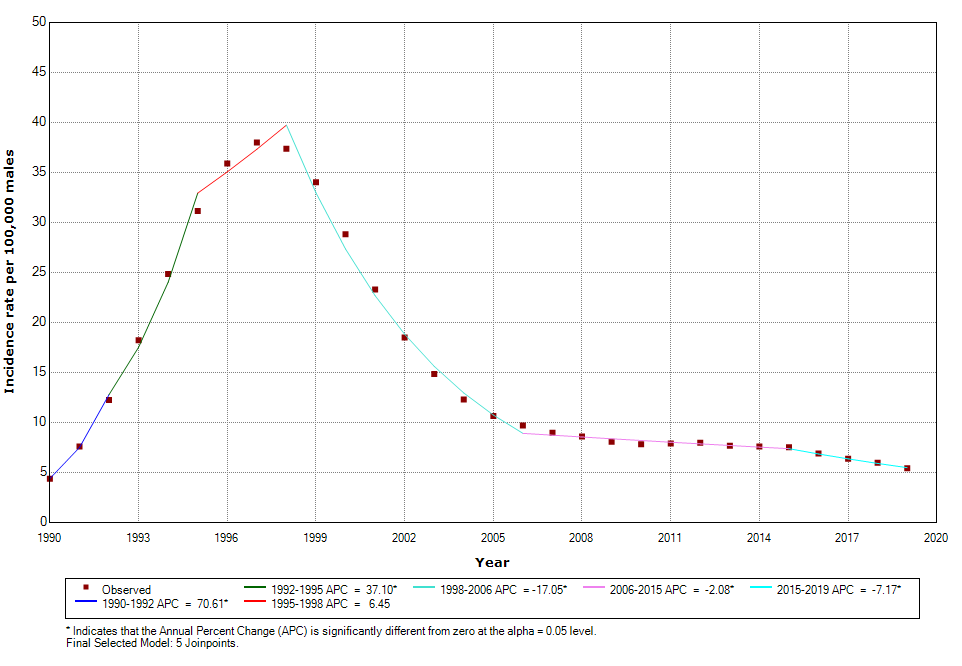 |  |
